# Exploring autistic traits in parents of autistic children: a pilot study on the broader autism phenotype

**DOI:** 10.3389/fpsyt.2025.1537487

**Published:** 2025-03-13

**Authors:** Antonio Narzisi, Elena Maria Busuoli, Maddalena Fabbri-Destro, Martina Pinzino, Sara Calderoni, Raffaella Tancredi, Michael Vincent Lombardo, Gabriele Masi

**Affiliations:** ^1^ IRCCS Stella Maris Foundation, Pisa, Italy; ^2^ Laboratory for Autism and Neurodevelopmental Disorders, Center for Neuroscience and Cognitive Systems, Istituto Italiano di Tecnologia, Rovereto, Italy; ^3^ IN-CNR Neuroscience Institute, Parma, Italy; ^4^ Department of Clinical and Experimental Medicine, University of Pisa, Pisa, Italy

**Keywords:** autism (ASC), broader autism phenotype (BAP), children, parents, offspring

## Abstract

**Background:**

Biological parents of autistic children often exhibit characteristics associated with the broader autism phenotype (BAP), a subclinical expression of autism-related traits. While BAP traits are known to be prevalent among first-degree relatives of autistic individuals, the relationship between parental traits and children’s characteristics remains under-explored.

**Objectives:**

This pilot study examines the presence and extent of BAP traits in an Italian sample of both biological mothers and fathers of autistic children, exploring also the correlations between parental BAP traits and children's autistic traits.

**Methods:**

Seventy-six autistic children (ages 4-11) and their biological parents were recruited for the study. Parental and child traits were evaluated using the Autism Quotient (AQ) and the Autism Diagnostic Observation Schedule-Second Edition (ADOS-2).

**Results:**

Of the recruited sample of parents, 29% fell within the BAP, with 12% of fathers and 17% of mothers meeting the criteria. A significant difference was found in AQ scores between fathers and mothers, with fathers scoring higher on average. Fathers' AQ scores were significantly correlated with their children's AQ scores, while no significant correlation was observed between mothers' AQ scores and children's scores. Additionally, children's AQ scores correlated significantly with all Vineland Adaptive Behavior Scale (VABS) subscales but not with ADOS scores.

**Conclusions:**

This study highlights the significance of parental BAP traits in relation to autism characteristics in children. The findings suggest that paternal BAP traits, in particular, may have a stronger association with child characteristics. Understanding these associations may contribute to refining psycho-educational strategies tailored to the specific traits of parents, ultimately enhancing intervention approaches.

## 1 Introduction

Research has shown that parents of autistic children often exhibit social characteristics similar to those observed in autistic individuals (1, 2), thought these typically do not impair daily functioning to the same extent as in clinically diagnosed individuals. The presence of subclinical traits resembling autism within families of affected individuals is known as the broader autism phenotype (BAP) (3–9). Over the past three decades, studies have reported a significant prevalence of BAP traits among first-degree relatives of autistic individuals compared to the general population (10–12). Reported BAP prevalence in parents of autistic children varies widely, with rates ratings from 3.0% (9) to 52.0% (6, 13) in mothers, and from 2.6% (9) to 80.0% (13, 14) in fathers. When considering both parents, prevalence ranges from 5.3% to 56.0% (15, 16). While BAP is often studied in the context of families with an autistic member, it is also observed in the general population, albeit at lower prevalence rates. This distinction is important for framing BAP not only as a family-specific phenomenon but also as a broader, population-wide trait.

BAP traits encompass various social and communication difficulties, including pragmatic challenges, reduced social skills, rigidity, stereotyped behaviors, impaired emotional recognition, and emotional aloofness (17–19). In adults, BAP traits have been linked to psychological conditions like anxiety, depression, and obsessive-compulsive disorder, as well as cognitive features including weak central coherence, reduced executive functioning, and altered neurological processing (7, 19). Investigating the BAP in parents is crucial due to the considerable contribution of hereditary and genetic factors in the etiology of autism (20). The co-occurrence of BAP traits in parents and the autism in children suggests potential genetic mechanisms (21). Adopting a stratified approach based on familial autism predisposition may help reduce genotypic and phenotypic heterogeneity in autism research (7, 19).

Initial BAP studies primarily involved qualitative family assessments (22), but later evolved to include structured psychometric scales (23–25). Initially, BAP traits were assessed using dichotomous measures (19), but since the 2000s, research has increasingly conceptualized BAP as a continuous, dimensional phenomenon rather than a binary classification (14, 23, 26). This dimensional perspective aligns with the ’spectrum’ approach to autism (27), framing BAP as a continuum of traits within both the general population and biological relatives of autistic individuals. Consequently, psychometric scales have been developed to measure these traits more precisely (19, 28–30).

Consistent findings indicate higher BAP prevalence in parents of autistic children compared to the general population (31, 32); although some studies have not replicated this association (33). A significant gap remains in understanding whether parental BAP traits increase the likelihood of autism in offspring or correlate with greater severity of symptoms in diagnosed children (34). Additionally, few studies examine the relationship between parental traits and symptomatology in autistic children (35).

Previous studies have suggested that fathers may exhibit higher BAP scores than mothers, particularly in the domains of social aloofness and rigidity, though findings remain inconsistent (26, 36). While some research has found a positive correlation between parental BAP and child autistic traits, particularly in fathers (30, 34), other studies have reported weaker or non-significant associations (37, 38). Given that BAP traits seem to be unequally distributed between mothers and fathers of autistic children (39), one remaining open question concerns potential sex differences in parental BAP expression.

Beyond parental influences, it remains unclear how child characteristics, such as cognitive ability, interact with autistic traits and adaptive functioning. Prior studies indicate that autistic traits may be more pronounced in individuals with higher cognitive abilities (16), while others suggest that adaptive functioning deficits may be more severe in children with lower IQ (40). Additionally, adaptive functioning, which refers to an individual's ability to manage daily life demands and social responsibilities, plays a vital role in understanding how autistic traits manifest in different children (41). Investigating these child-specific factors, alongside parental BAP traits, may provide deeper insight into the complex interplay between genetic predisposition and developmental outcomes in autism.

This background informs our pilot study, which aims to assess the relationship between parental BAP traits and symptom severity of autistic children. The objectives of the study are 1) to evaluate the presence and extent of BAP in an Italian sample of parents of autistic children using the self-report Autism Quotient (AQ) scale; 2) investigate correlations between the autistic traits of parents and the autistic traits of their autistic children; 3) describe in autistic offspring the correlations between autism severity and adaptive level, taking into account differences in cognitive level.

Based on existing literature, we hypothesize that fathers of autistic children exhibit a higher prevalence of BAP traits compared to mothers and that fathers' AQ scores are more strongly correlated with those of their children (30, 34). Additionally, we predict that autistic traits in children will be inversely associated with their adaptive abilities, particularly in the domains of socialization and daily living skills (41). Finally, we expect that the correlations between parental BAP traits and child characteristics will vary according to the child's cognitive level, with more pronounced effects in children with higher IQs (40).

## 2 Method

### 2.1 Sample

Participants were recruited at the IRCCS Stella Maris Foundation in Calambrone (Pisa). Inclusion criteria were the following: (a) age between 4 and 11 years; (b) autistic children according to DSM-5 criteria (42); (c) ADOS-2 (Autism Diagnostic Observation Schedule; 43) positive for autism. Based on available information, exclusion criteria were: (a) presence of neurological syndromes or focal neurological signs; (b) significant sensory impairments; (c) history of birth asphyxia, premature birth, head trauma, epilepsy; (d) use of antipsychotic drugs. Based on these criteria, 76 children, including 13 females and 63 males, were consecutively recruited (mean age 7,21 ± 1,95 years) (**Table 1**). Power analysis at this sample size (n=76) indicates that the minimum effect size needed for a dependent samples t-test with 80% power and an alpha of 0.05 would be d = 0.34. For a Pearson correlation analysis, the minimum r statistic would need to be 0.33 or higher to achieve power of at least 80% at an alpha of 0.05. For the IQ subgroup analysis, the sample size is split roughly in half and this implies that minimum effect sizes would be much higher for similarly powered tests (e.g., d = 0.49 for a t-test, r = 0.45 for a Pearson correlation).

**Table 1:** Clinical characteristics of autistic children (N=76).

	Mean (SD)
ADOS-CSS	5,49 ± 2,02
AQ - Total	79,39 ± 22,29
FSIQ (Wechsler scales)	91,62 ± 19,22
VABS-2 - Communication	77,25 ± 29,06
VABS-2 - Daily Living	58,92 ± 22,24
VABS-2 - Socialization	48,5 ± 18,79
VABS-2 - Composite	66,79 ± 16,21

ADOS-CSS, Autism Diagnostic Observation Schedule-Calibrated Severity Score; AQ, Autism Quotient; FSIQ, Full Scale Intelligent Quotient; VABS-2, Vineland Adaptive Behaviour Scales-2 Edition.

Considering parents, the inclusion criteria were: (a) having only one autistic child, aged between 4 and 11 years (single-incidence/simplex families - SPX), and (b) being biological parents. A total of 152 biological parents were included: 76 fathers (mean age 44.79 ± 6.74 years; age range 32 to 69 years) and 76 mothers (mean age 41.41 ± 4.88 years; range age 29 to 54 years).

#### 2.1.2 Ethical statement

The data analyzed in this study were collected during routine clinical visits and not specifically for research purposes. In compliance with General Data Protection Regulation (GDPR) requirements, the study ensured that all data were fully anonymized prior to analysis, preventing participant identification. The anonymization process involved the removal or encryption of personally identifiable information, adhering to established data protection standards. Given that the study utilized existing clinical data without additional interventions or risks to participants, ethical committee approval was not required. However, the study adhered to ethical and legal guidelines for handling clinical data, including transparency through clear policies and compliance with applicable data protection regulations. Additionally, all procedures followed the ethical principles outlined in the Declaration of Helsinki.

### 2.2 Measures

All instruments used in the research were administered or self-completed during the hospitalization.

Autism-Spectrum Quotient (AQ), adult version, is a self-administered questionnaire developed by Simon Baron-Cohen et al. (44) that assesses the presence and extent of autistic traits in individuals aged ≥ 16 years with IQs in the normal range. It is a screening tool consisting of 50 items on a continuum from clinically significant autistic traits to typical development (45). It comprises five subscales of 10 items each: Social skills, Attention Switching, Attention to detail, Communication, and Imagination. The subject is asked to respond to a series of statements, choosing from 4 options: absolutely agree, partially agree, partially disagree, absolutely disagree. The total score on the AQ is given by the sum of the scores obtained at each item. Here, we used the Italian version of the AQ (25) in which the cut-offs of the autism phenotype are Broader Autism Phenotype (BAP), 21-27 (AQ score 1 to 2 SD above the mean), Medium Autism Phenotype (MAP), 28-32 (AQ score 2 to 3 SD above the mean), Narrow Autism Phenotype (NAP) ≥ 32 (AQ score ≥ 3 SD above the mean). A subject with scores >21 is included in the broad autism phenotype (BAP).

Autism-Spectrum Quotient-child (AQ-child) (46) is a parent-report questionnaire measuring the extent of autistic traits in children aged 4-11. It consists of 50 items subdivided into five subscales of 10 items each: Social skills, Attention Switching, Attention to detail, Communication, and Imagination. The AQ-child has shown good sensitivity (95%), good specificity (95%), good test-retest reliability, and high internal consistency (46). One of the parents fills out the questionnaire and is asked to respond to the proposed statements by considering the child's characteristics and choosing one of 4 options: strongly agree, partially agree, partially disagree, or strongly disagree. The scores obtained for each item give the total score for the AQ-Child. The range of scores is from 0 to 150. The minimum score of 0 indicates the total absence of autistic traits, while at the opposite extreme, the maximum score of 150 indicates the full presence of autistic traits. Therefore, the higher the child's score, the greater the presence of autistic traits.

*Autism Diagnostic Observation Schedule-Second Edition (ADOS-2)* (43) is a semi-structured, standardized observation protocol for assessing a child's behavior in interaction with an examiner.

Vineland Adaptive Behavior Scales-2 (VABS-2) (47, 48) assesses, through a semi-structured interview directed at the parent, the adaptive behavior regarding personal autonomy and social responsibility. The VABS-2 was included in this study to explore the relationship between autistic traits and adaptive functioning in autistic children. While not a direct measure of autistic traits, adaptive behavior has been shown to be significantly associated with specific support needs of autistic children (49).

### 2.3 Data analysis

A paired-sample t-test was used to evaluate differences between mother and father in AQ scores. Standardized effect size (Cohen’s d) was used to describe the size of the difference. Pearson’s correlation was used to test child and parent AQ, and Fisher’s z-test was used to find confidence intervals for r and differences between correlations. Pearson’s correlations were also computed for child AQ, VABS, and ADOS-CSS scores. Similarly, correlations were tested within IQ subgroups defined based on a median split of full-scale IQ scores (median FIQ = 89). The Mantel test examined the correlation between high and low-IQ subgroups. Finally, BAP, MAP, and NAP subgroups were defined using mean and standard deviation norms from Wheelwright et al. (26). Where possible, we also substituted linear mixed effect modeling instead of the data analyses described above, as a secondary check on the results with a different modeling technique.

## 3 Results

### 3.1 AQ: t test mothers’ versus fathers’ scores

**Figure 1:**
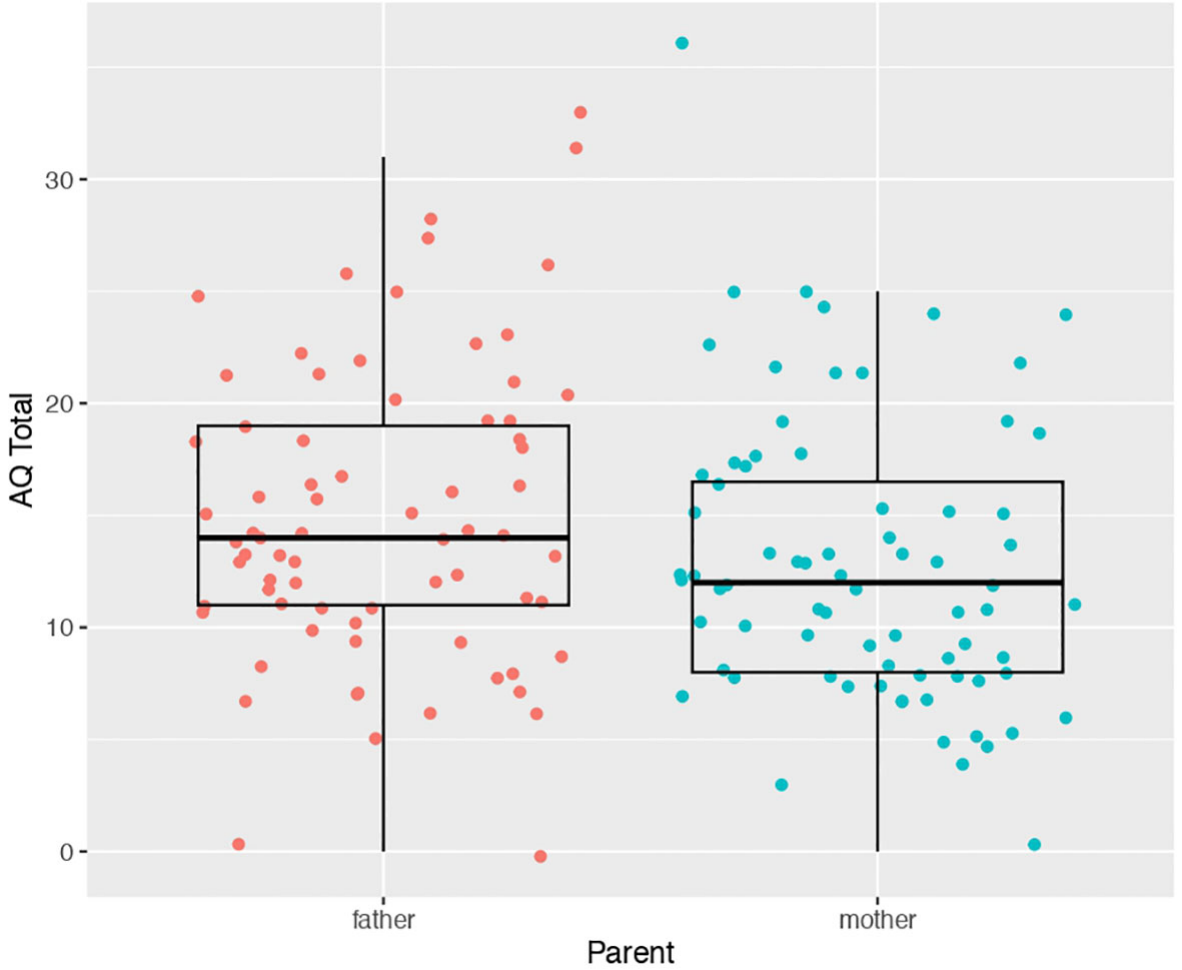
Scatter-boxplot of AQ scores for mothers (blue) versus fathers (pink) of autistic children.

Our first analysis compared AQ scores between mothers (mean score 12.78 ± 6.36) and fathers (mean score 14.87 ± 1.80) of autistic children. We found a statistically significant difference (t(71) = -2.2013, p = 0.03097) between father vs mother AQ scores. Mothers had slightly lower AQ scores than fathers on average (d = -0.31) (**Figure 1**). A similar result is obtained if the analysis employed was a linear mixed effect model with AQ as the dependent variable, mother vs father status as a fixed effect, and family ID as a random effect modeled with random intercepts (t = -2.21, p = 0.03).

### 3.2 Correlations between parents’ AQ and children’s AQ

**Figure 2:**
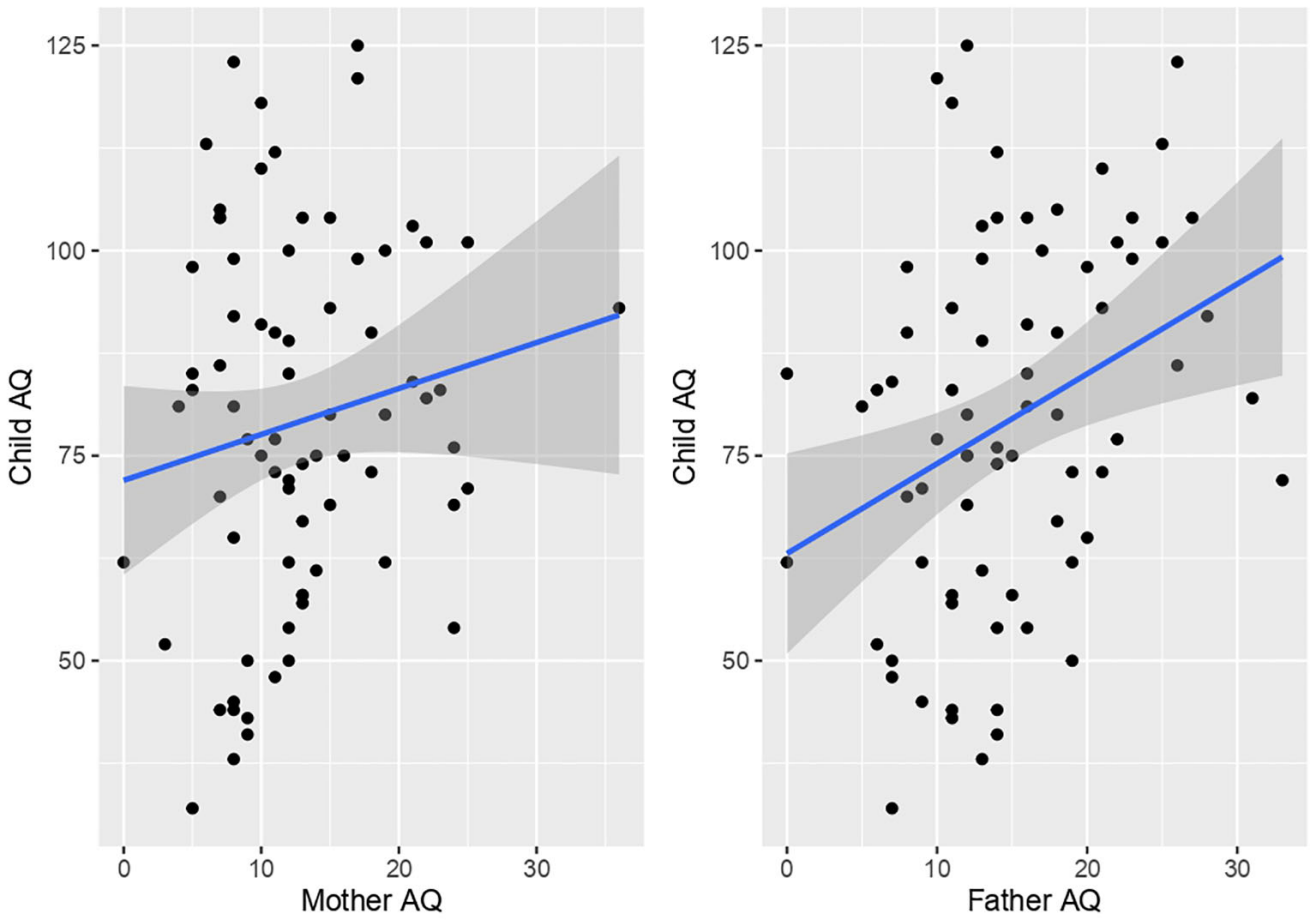
Scatterplots of child AQ (y-axis) by parent AQ (x-axis).

Child AQ total was significantly positively correlated with father’s AQ total (r(71) = 0.32, p = 0.004). In contrast, no significant correlation was present between child AQ total and mother AQ (r(73) = 0.15, p = 0.1714) (**Figure 2**). However, significant differences between correlations of child with mother AQ versus child and father AQ were not present (z = 1.06, p = 0.29). Comparison of correlations between mother versus father AQ scores indicated a trend for a positive association (r(70) = 0.23, p = 0.05064).

### 3.3 Tests of correlation between child AQ and VABS and ADOS-CSS

**Figure 3:**
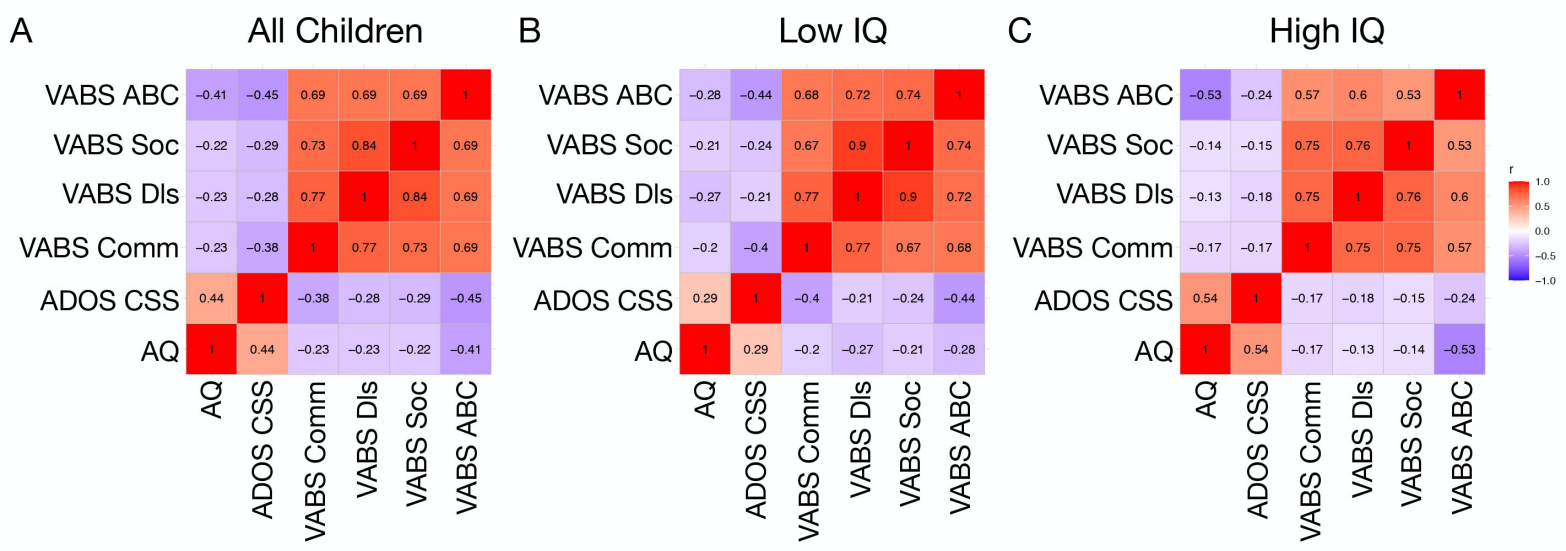
Correlation matrices showing associations between AQ, ADOS CSS, and VABS domain scores in all children **(A)**, a low IQ **(B)**, and high IQ **(C)** subgroup.

From the full sample of all autistic children, we examined correlations between AQ, VABS, and ADOS CSS scores. This analysis identified significant negative correlations between AQ and VABS domains (r<-0.29, p <0.02). A positive correlation at trend level significance was also observed between AQ and ADOS CSS scores (r = 0.44, p = 0.056) (**Figure 3A**).

### 3.4 Tests on subgroups after median split on IQ

Similar correlations between child AQ and VABS or ADOS CSS scores were examined within IQ subgroups defined on the basis of a median split of full scale IQ. In the low IQ group, AQ was negatively correlated with VABS domains (r<-0.20, p<0.04). However, AQ was not significantly correlated with ADOS CSS (r = 0.29, p = 0.18) (**Figure 3B**). In contrast, within the high IQ group there were significant negative correlations between AQ and VABS domains (r<-0.13, p< 0.037) as well as a significant positive correlation between AQ and ADOS CSS (r = 0.54, p = 0.039). Comparison of the correlation matrices between the low and high IQ subgroups revealed significant similarity between the two subgroups, indicating that the correlations are not significantly dissimilar across AQ, ADOS CSS, and VABS (Mantel’s r = 0.83, p = 0.013) (**Figure 3C**).

We next re-ran analyses to see if mother versus father status interacts with child IQ subgroup in predicting AQ scores. This analysis tests whether the differences shown in **Figure 1** are different depending on the child’s IQ subgroup. The model run here is the same linear mixed effect model reported at the beginning of the Results section, but now includes the child’s IQ subgroup variable as well. The effect of subgroup did not significantly interact mother vs father status (parent*subgroup F = 3.3, p = 0.07), while the effect of parent (e.g., mother vs father) was still significant (parent F = 4.71, p = 0.033), and the main effect of child’s IQ subgroup was not significant (subgroup F = 0.49, p = 0.48). Overall, this result indicates that differences between mother and father AQ scores is not heavily changed as a function of the child’s IQ subgroup. Furthermore, parent AQ scores are similar in both child IQ subgroups.

We also re-ran analysis that attempts to predict the child’s AQ and based on mother or father’s AQ and stratified by IQ subgroup. The models here treated child AQ as the dependent variable and had parent’s AQ and child IQ subgroup as independent variables, while also crucially testing the parent*subgroup interaction effect. Here we find that parent*subgroup interactions were not statistically significant for predicting the child’s AQ score for mothers (F = 0.30, p = 0.57) or for fathers (F = 0.14, p = 0.70). Thus, similar relationships exist between the child’s AQ score and the parent’s IQ score regardless of the child’s IQ subgroup label.

### 3.5 BAP, MAP, and NAP AQ subgroups in mothers and fathers

In a final analysis, we examined BAP, MAP, and NAP AQ subgroup distinctions as defined by norms provided in Wheelwright et al. (26). Here we find that the large majority of all the parents in this sample do not heavily conform to BAP, MAP, or NAP subgroups. In particular, 85% of mothers fall outside of the BAP, MAP, or NAP zones. Similarly, 89% of the fathers would not fit under the BAP, MAP, and NAP criteria outlined by Wheelwright et al. (26). However, 17% of mothers (n=11) and 12% of fathers (n=8) are in the BAP.

## 4 Discussion

**Figure 4:**
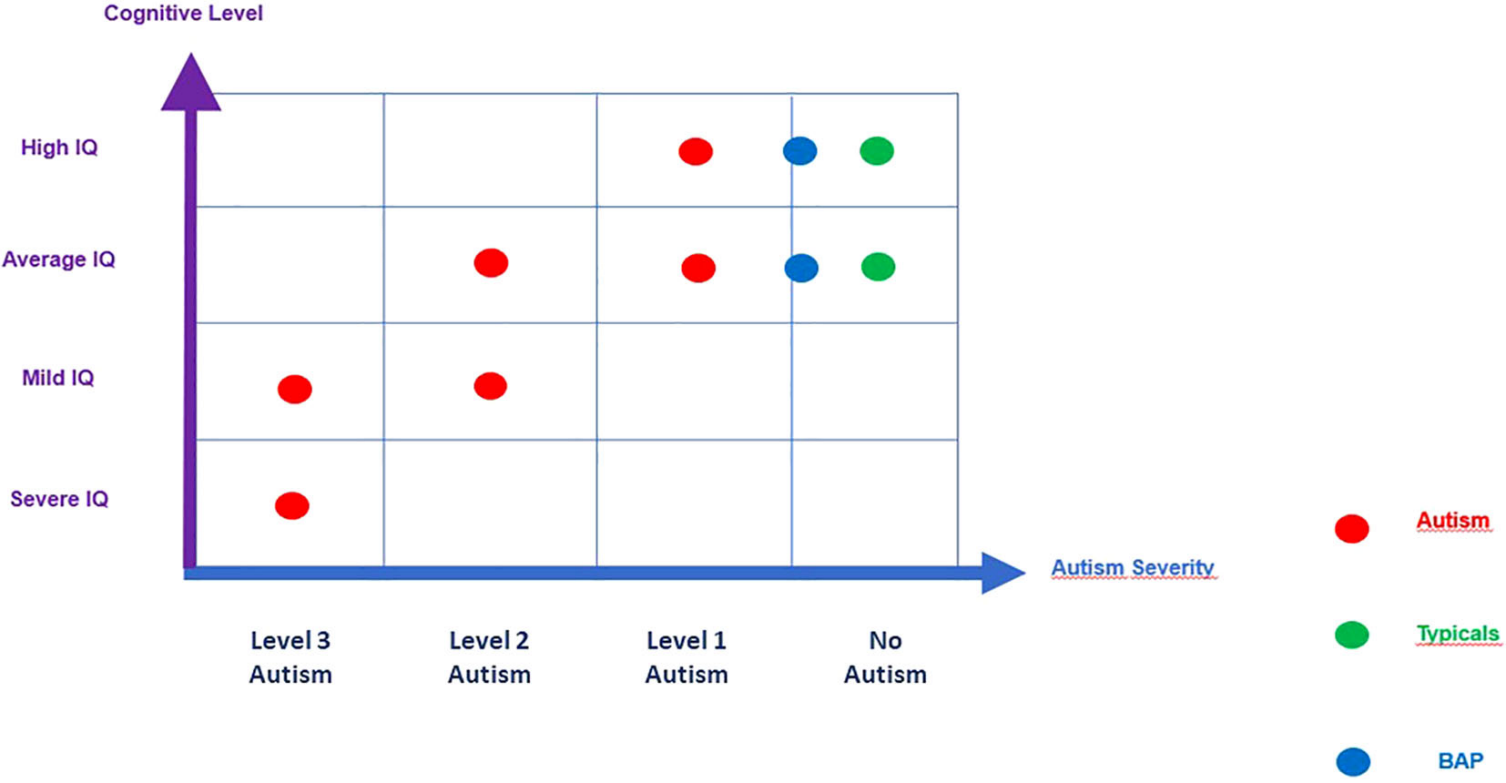
Iconographic representation of the Broader Autism Phenotype.

The Broader Autism Phenotype (BAP) is characterized by subclinical traits that are observed in parents of autistic children despite the absence of a formal diagnosis and typically normal or above-average cognitive level (**Figure 4**). Our study highlights statistically significant differences between the Autism Quotient (AQ) scores of fathers and mothers of autistic children. This finding diverges from Wheelwright's (26) and Kose (29), who did not report significant gender differences in BAP traits. Specifically, our results suggest that fathers exhibit more pronounced traits associated with autism than mothers when evaluated alongside their children (50).

Several studies have investigated the presence and nature of autistic traits in parents of autistic children, supporting the notion that these characteristics are heritable and part of a broader phenotype. Earlier research by Piven et al. (51) demonstrated that parents of autistic children exhibit higher rates of rigid personality traits and difficulties in social communication compared to control groups. Similarly, Bishop et al. (30) found that parents of autistic children score significantly higher on measures of pragmatic language impairments, reinforcing the hypothesis that BAP traits may reflect a genetic liability to autism.

Our results align with Lyall et al. (52), who employed the Social Responsiveness Scale (SRS) and found similarities in social traits between fathers and their sons, whereas mother-son pairs did not exhibit the same correlation. Similarly, Situ et al. (53) reported a significant correlation between fathers’ AQ scores and those of their autistic children. These findings underscore the notion that certain subclinical traits may be quantitatively inherited, suggesting that the degree of a child’s autistic condition correlates with the presence of sub-threshold autistic traits in their fathers (23, 26, 44, 53). Notably, studies such as those by Seidman et al. (14) and Losh et al. (9) indicate that fathers may exhibit more pronounced BAP traits than mothers, although maternal traits still play a significant role in shaping social and communicative development in children.

In our sample of autistic children, we observed significant correlations between the child’s AQ and the Vineland Adaptive Behavior Scales (VABS), reinforcing the importance of adaptive behavior assessment in autistic individuals, with particular attention to the Socialization scale's inverse relationship with specific support needs (54). Corroborated by previous research by Klin (55) and Perry (56), our findings suggest that greater autistic traits in children are associated with deficits in adaptive behavior.

When examining the sample according to IQ, we found that the low IQ group did not exhibit significant correlations between AQ scores and clinical assessments from the ADOS and VABS. This aligns with findings from Auyeung et al. (46), suggesting that adaptive behavior may be more closely tied to cognitive needs, particularly in autistic children. Differences between levels of autism emerged only in the Vineland scales, further emphasizing the impact of intellectual capacity on adaptive behavior outcomes (48).

Our analysis of parents with AQ scores indicating BAP traits showed that most parents did not meet the criteria for BAP, with only 12% of fathers and 17% of mothers classified as such. Interestingly, our study found a higher prevalence of BAP traits among mothers compared to fathers, contrasting with findings by Möricke et al. (57), which reported greater similarities between mothers’ traits and those of their autistic children than between fathers’ traits and their children’s.

Despite the relatively low percentage of parents exhibiting BAP characteristics, detecting these traits in the parents of autistic children is crucial for understanding the multifactorial etiology of autism. Identifying BAP traits can inform the development of specialized psycho-educational strategies for parents. Tailored interventions, such as parent-coaching methodologies, can be designed to leverage the shared traits between parents and children, thus enhancing therapeutic outcomes. In this context, paraphrasing Archimedes, these 'similarities' could serve as a valuable foundation for effective therapeutic interventions.

## 5 Study’s limitations

While the present study provides valuable insights into the Broader Autism Phenotype (BAP) in parents of autistic children, several limitations must be acknowledged. First, the study lacks a control group of parents from the general population, limiting our ability to compare BAP traits between parents of autistic children and those without autism. Additionally, the sample size is relatively small and not fully representative of broader populations, warranting caution when generalizing the findings. Given the lower statistical power and higher minimum effect sizes needed for sufficiently powered tests in the IQ subgroups analyses, caution needs to be emphasized when interpreting the non-significant differences between subgroups. Future work with much larger sample sizes that allow for higher statistical power may be needed to detect possible true, yet more subtle effects in this type of analysis. Moreover, the sample consisted predominantly of male children, which may have influenced the findings, particularly in relation to parental BAP traits and their associations with child characteristics. This gender imbalance, combined with the small sample size, limits the generalizability of the results. Future research should aim to include more diverse samples in terms of both child gender and family composition. Additionally, it would be valuable to explore the potential effects of different parent-child relationships (e.g., father-son, mother-son, father-daughter, mother-daughter) to better understand how parental BAP traits may vary across these dyads.

Second, the study relies solely on parental self-reports using the Autism Quotient (AQ) scale, which, while widely used, may not fully capture the complexity of BAP traits. Given that questionnaires are subjective measures, parental responses may be influenced by personal perceptions of their child’s traits. This introduces a potential bias that could affect the accuracy of reported BAP characteristics. It is also important to note that the AQ has been shown to have limitations in detecting autistic traits in non-clinical populations (58, 59), and while the AQ remains a valuable tool in autism family studies, it may not fully reflect the broader autism phenotype in all contexts. Future studies should consider incorporating multi-informant assessments or analytical controls to mitigate this limitation. Additionally, including other psychometric tools, such as the Social Responsiveness Scale (SRS), or observational methods that assess social and communicative impairments (23, 59), could provide a more comprehensive understanding of these traits.

Moreover, the study does not account for socio-economic factors that may influence the expression of BAP traits in parents. Variables such as education level, income, and access to healthcare could contribute to the observed patterns and should be considered in future research. Additionally, environmental factors, including familial stress, should be explored further, as they may play a role in shaping both parental and child autistic traits (60).

Finally, the cross-sectional nature of the study prevents causal inferences. Longitudinal studies tracking parental BAP traits over time, along with their impact on child development, would be beneficial. Future research should also aim for larger and more diverse samples to improve the generalizability of findings and provide a clearer picture of how genetic and environmental factors interact in shaping the broader autism phenotype.

## Data Availability

The raw data supporting the conclusions of this article will be made available by the authors, without undue reservation.
